# Peeling back the layers: Unveiling the biochemistry of health-promoting molecules in orange fruits

**DOI:** 10.1093/plphys/kiad272

**Published:** 2023-05-10

**Authors:** Henryk Straube

**Affiliations:** Assistant Features Editor, Plant Physiology, American Society of Plant Biologists, USA; Faculty of Science, Department of Plant and Environmental Sciences, Section for Plant Biochemistry, University of Copenhagen, 1871 Frederiksberg, Copenhagen, Denmark

Orange trees (*Citrus sinensis*) are plants from the genus Citrus and an economically important fruit crop (FAO 2021). Orange trees were likely first cultivated in China and northeast India before being introduced to Europe in the 11th century. At that time, orange fruits were quite bitter and were probably grown for their medicinal properties. Later, Europeans brought the orange tree to South America (Milind and Dev 2012). Orange trees produce, like other plants, specialized metabolites with functions as allelochemicals that inhibit the growth of other plants, drive plant–microbe interactions, or serve as defense chemicals (Weng et al. 2021).

Flavonoids are a group of specialized metabolites that have been shown to contribute to all the aforementioned aspects (Weston and Mathesius 2013). Polymethoxylated flavonoids (PMFs) are a subgroup of flavonoids that are abundant in Citrus fruit peels and leaves (Figure). Researchers have identified up to 32 different PMFs in orange fruits. The most common PMFs include nobiletin, sinensetin, 6-demethoxytangeretin, heptamethoxyflavone, tangeretin, and 5-demethoxynobiletin (Xing et al. 2017). PMFs are stored in epicuticular waxes and likely serve as antimicrobial and antifungal defense compounds (Johann et al. 2007). Besides their role in plant physiology, PMFs have promising properties for medical application (Milind and Dev 2012; Zhao et al. 2020).

**Figure.**
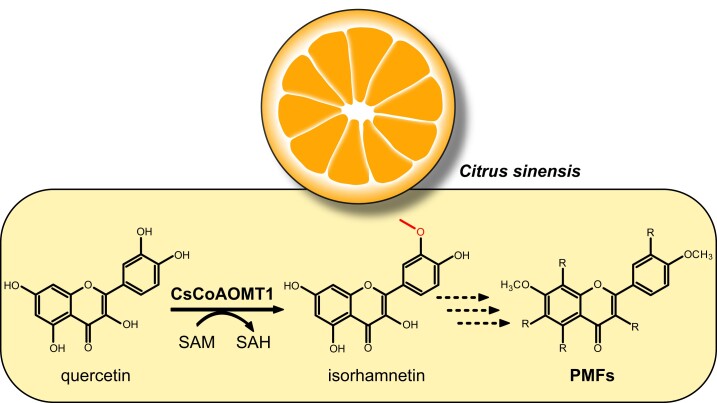
A schematic overview of the involvement of CsCoAOMT1 in the biosynthesis of PMFs in orange fruit flavedo. The SAM-dependent O-methyltransferase characterized by Liao et al. (2023) promotes the accumulation of PMFs in orange fruits, likely by methylating vicinal hydroxyl groups of flavonoid precursor molecules at the 6-, 7-, 8-, and 3′- position. As an example, the methylation of quercetin to isorhamnetin by CsCoAOMT1 is shown using SAM as a methyl group donor. The dotted arrows depict potential hydroxylation and methylation reactions leading to the formation of PMFs in orange fruit flavedo. R can be an H, OH, or OCH_3_, depending on the type of PMF. SAH, S-adenosyl homocysteine.

Flavonoids derived from the phenylpropanoid pathway serve as precursors for PMF biosynthesis in plants. Flavonoids are oxygenated and methylated by enzymatic reactions to yield PMFs (Fig.; Liao et al. 2023). Methylation of flavonoids is usually catalyzed by S-adenosyl-L-methionine (SAM)-dependent O-methyltransferases (OMTs; Berim et al. 2012), which can be grouped into 2 subfamilies: the Mg^2+^-dependent caffeoyl coenzyme A OMT (CoAOMT) subfamily and the caffeic acid OMT (COMT) subfamily (Joshi and Chiang 1998).

In this issue of *Plant Physiology*, Liao et al. (2023) demonstrate that CsCoAOMT1 is involved in the biosynthesis of PMFs in oranges (Figure). To identify candidate genes participating in PMF biosynthesis, the authors chose 70 potential OMT genes that showed expression in the flavedo, the outermost, orange-colored layer of the fruit of *C. sinensis* “Bingtangcheng.” The genes were grouped into 63 *COMT* and 7 CoAOMT candidate genes by performing a phylogenetic analysis and comparing the respective amino acid sequences for conserved domains. Because previous studies on PMF biosynthesis in Citrus focused mostly on the COMT subfamily proteins and could not explain all identified PMF methylation sites, the researchers decided to study the CoAOMT subfamily proteins. Analyzing the content of PMFs in the flavedo during different fruit ripening stages the researchers showed that sinensetin and nobiletin are the most abundant PMFs in the milligram per gram fresh weight range. In total, 6 PMFs were shown to be abundant during all studied ripening stages (Figure). Comparing PMF abundance and the expression pattern of the *CoAOMT* candidate genes in the same tissues, Liao and colleagues decided to analyze *CsCoAOMT1* for its involvement in PMF metabolism.

To determine whether the identified gene has O-methyltransferase activity on flavonoids, the authors tested the recombinant CsCoAOMT1 for activity with different phenolic substrates using SAM as a methyl group donor. CsCoAOMT1 showed activity with several flavanones, flavones, flavonols, caffeoyl-CoA, and esculetin. CsCoAOMT1 did not methylate the flavone apigenin and the flavonol kaempferol, presumably because they lack vicinal hydroxy groups, and the enzyme did not methylate caffeic acid and gallic acid. CsCoAOMT1 was then analyzed for its kinetic parameters, uncovering that it has the highest catalytic efficiency toward the flavonol quercetin. The enzyme showed a lower catalytic efficiency with other tested flavones and flavanones. Noteworthy, the enzyme had a comparatively low catalytic efficiency toward the CoAOMT characteristic substrate caffeoyl-CoA (K_cat_/K_M_ = 128.9 M^−1^s^−1^).

Because other CoAOMTs have mostly been studied and shown to methylate a wide range of substrates, the authors analyzed the function of CsCoAOMT1 in vivo. Transient overexpression of *CsCoAOMT1* in orange peels led to an increased transcript abundance of *CsAOMT1*. In line with this result, the concentration of the 6 major PMFs—isosinensetin, sinensetin, nobiletin, 6-demethoxytangeretin, heptamethoxyflavone, and tangeretin—significantly increased compared with the control treatment. Virus-induced gene silencing in *C. sinensis* “Jincheng” seedlings showed that downregulation of CsCoAOMT1 transcript leads to a significantly lower concentration of 5 major PMFs: sinensetin, nobiletin, 6-demethoxytangeretin, heptamethoxyflavone, and tangeretin.

In summary, Liao et al. (2023) provide evidence that CsCoAOMT1 is a multifunctional O-methyltransferase involved in the biosynthesis of PMFs in oranges. In vitro, the enzyme is capable of converting 6-, 7-, 8-, and 3′-OH of flavonoids with vicinal hydroxyl groups to the corresponding monomethyl ethers and methylating certain nonflavonoid substrates such as esculetin or caffeoyl-CoA (Figure). Changes in PMF abundance in orange plants upon modulation of *CsCoAOMT1* transcript abundance further underline the involvement of the enzyme in PMF biosynthesis. These findings will improve our understanding of the biosynthesis pathway of PMFs in plants, molecular breeding approaches, and biotechnological production of antimicrobial and antifungal PMFs by pathway reconstruction in heterologous systems.
